# Lower-Segment Transverse Cesarean Section

**DOI:** 10.1055/s-0040-1708060

**Published:** 2020-06-09

**Authors:** Yuji Hiramatsu

**Affiliations:** 1 Okayama City General Medical Center, Kita-Ku, Okayama, Japan

**Keywords:** cesarean section, lower-segment transverse section, ultrasonography

## Abstract

Cesarean section is the ultimate method of successful delivery of infants under various circumstances and is an indispensable operation in obstetrics. However, the degree of difficulty varies greatly depending on the gestational weeks, number of fetuses, number of previous cesarean sections, degree of placental adhesion, presence of uterine myomas, maternal obesity, and other factors. In addition, emergency cesarean section is a battle against time, and prompt surgery is required.

During training in cesarean section, surgeons must master the basic techniques in cases of term head presentation first. They must then master the techniques in cases involving complications such as malpresentation, preterm birth, placenta previa, abruptio placentae, uterine myomas, and other conditions.

Cesarean section itself is a simple operation. However, there are many difficult cases, and many complications such as placenta accreta and defects of the incision scar may occur after cesarean section.

The present report describes the basic procedures and cautionary points to perform the cesarean section without complications.

## Indications for Cesarean Section

Two types of cesarean section are performed: elective and emergency cesarean section. The absolute indications and relative indications are as follows.

### Absolute Indications

Absolute indications are cephalopelvic disproportion, placenta previa, abruptio placentae, transverse lie, triplet pregnancy, mechanical obstruction of vaginal birth (large uterine myoma or ovarian tumor), prolapsed umbilical cord, vasa previa, human immunodeficiency virus-infected pregnancy, and other conditions.

### Relative Indications

Relative indications are nonreassuring fetal status, maternal complications (e.g., hypertensive disorder in pregnancy or cardiac disease), twin pregnancy, breech presentation, and other conditions.

## Classification by Dissection Method

Cesarean section may be classified as lower-segment transverse cesarean section or classical cesarean section (for a preterm infant, placenta previa, uterine myomas, etc.).

## Preoperative Examination and Preparation

### Maternal Examination

Blood tests, respiratory function tests, chest radiographs, electrocardiography, and urinalysis are performed preoperatively. Before entering the operating room, internal examination and vaginal douching are performed, and cervical dilation and the fetal station are confirmed. If the cervix is closed, Hegar dilators should be prepared for the dilatation of the cervical canal. If the cervical canal is dilated ≥5 cm, caution will be required at the time of low transverse incision as described later. The fetal station must also be confirmed because it is related to the degree of difficulty of fetal head delivery. If cesarean section is planned for breech presentation, the breech presentation may be corrected to the head presentation before the operation in rare cases. Therefore, it is necessary to reconfirm the fetal presentation with internal examination and ultrasonography immediately before cesarean section.

### Surgical Tips and Precautions

In cases of repeated cesarean section, it is important to collect information about the previous operation. During preoperative ultrasonography, the clinician should determine whether adhesion is present between the abdominal wall and uterine wall or whether the bladder has been lifted.

In addition to the normal operation record, another checklist is provided in our hospital. This checklist includes Bishop's score at the time of the operation, indication, status of the abdominal cavity, and points to keep in mind when performing the next cesarean section. This checklist is very useful for predicting the condition of the abdominal cavity at the time of the next surgery.

### Fetal Examination

Ultrasonography should be used to evaluate fetal lie, fetal body weight, location of the placenta, and amount of amniotic fluid.

### Complications and Informed Consent

Complications associated with cesarean section include massive hemorrhage, bladder injury, ureteral injury, intestinal tract injury, fetal injury (especially in the case of oligohydramnios), a sleeping baby (in patients undergoing general anesthesia), postoperative deep vein thrombosis, pulmonary embolism, wound dissection, and wound scarring. In addition, the degree of difficulty varies depending on the presence or absence of prior surgery (including cesarean section), obesity, maternal complications, and other conditions. Therefore, these conditions should be considered when obtaining informed consent.

## Explanation of Each Step 1 2 3 4 5

### Laparotomy

Methods of laparotomy include a lower abdominal midline longitudinal incision and transverse incision (Pfannenstiel incision). Both incisions should be of sufficient length to allow delivery of the fetus without difficulty.

### Bladder Peritoneal Incision

**Fig. 1:**
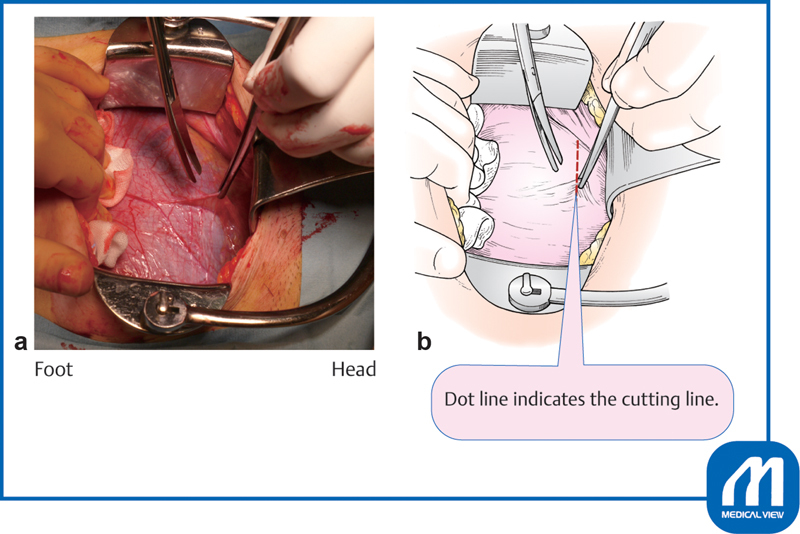
Bladder peritoneal incision and dissection of bladder. (Reproduced with permission of Hiramatsu Y. Lower-segment transverse cesarean section. In: Hiramatsu Y, Konishi I, Sakuragi N, Takeda S, eds. Mastering the Essential Surgical Procedures OGS NOW, No.3. Cesarean Section (Japanese). Tokyo: Medical View; 2010:28–41. Copyright © Medical View).

The urinary bladder is exposed and a transverse incision is made with Cooper scissors at the upper margin of the bladder, where the peritoneum is most roughly connected to the uterus **( Fig. 1 )** . The scissors are entered laterally and used to separate an approximately 2-cm-wide strip of serosa, which is then cut. The bladder is separated approximately 3 cm below the peritoneal incision line. Unnecessary dissection causes extra bleeding and subsequent adhesions.

### Lower-Segment Transverse Cesarean Section and Extension of the Incision

The lower-segment transverse uterine incision should be placed approximately 1 cm below the peritoneal incision. When an incision is made at a low position in the cervix, the uterine artery, uterine vein, and ureter are in close proximity to the outside of the incision. If the incision is lacerated laterally, problems easily occur and repair becomes difficult.

**Fig. 2:**
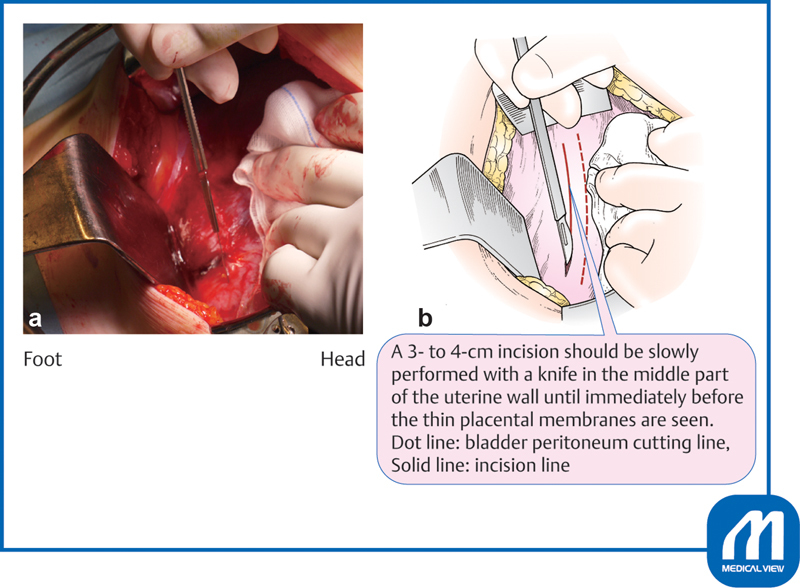
Lower uterine transverse incision and extension of the incision ①. (Reproduced with permission of Hiramatsu Y. Lower-segment transverse cesarean section. In: Hiramatsu Y, Konishi I, Sakuragi N, Takeda S, eds. Mastering the Essential Surgical Procedures OGS NOW, No.3. Cesarean Section (Japanese). Tokyo: Medical View; 2010:28–41. Copyright © Medical View).

**Fig. 3:**
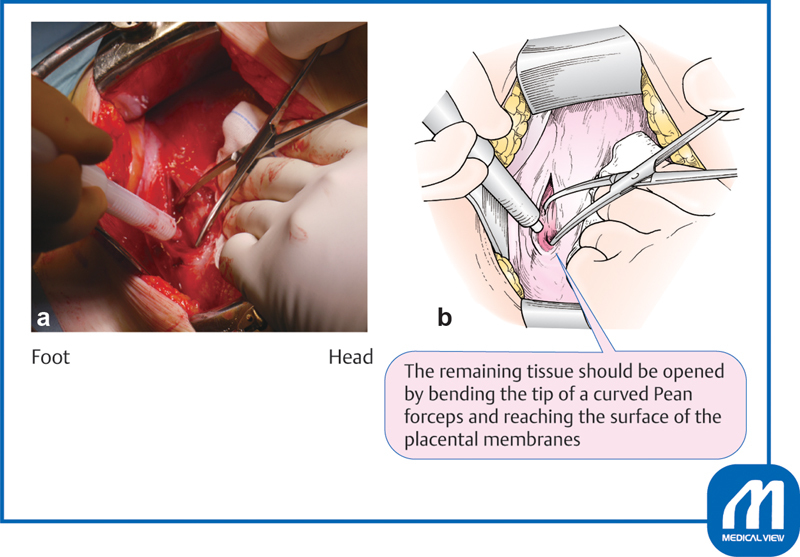
Lower uterine transverse incision and extension of the incision ②. (Reproduced with permission of Hiramatsu Y. Lower-segment transverse Cesarean section. In: Hiramatsu Y, Konishi I, Sakuragi N, Takeda S, eds. Mastering the Essential Surgical Procedures OGS NOW, No.3. Cesarean Section (Japanese). Tokyo: Medical View; 2010:28–41. Copyright © Medical View).

When performing a uterine muscle incision, the uterine wall should be extended upward and the bladder is squeezed downward to expose the incision site. A 3- to 4-cm incision should be slowly performed with a knife in the middle part of the uterine wall until immediately before the thin placental membranes are seen ( Fig. 2 ). Continuous suction by an assistant is very important at this time. The assistant should aspirate blood so that the bottom of the incision is clearly visible. Incision with a scalpel near the placental membrane increases the risk of injury to the fetus; therefore, the remaining tissue should be opened by bending the tip of a curved Pean forceps and reaching the surface of the placental membranes. This procedure is important to avoid injury to the fetus ( Fig. 3 ).

**Fig. 4:**
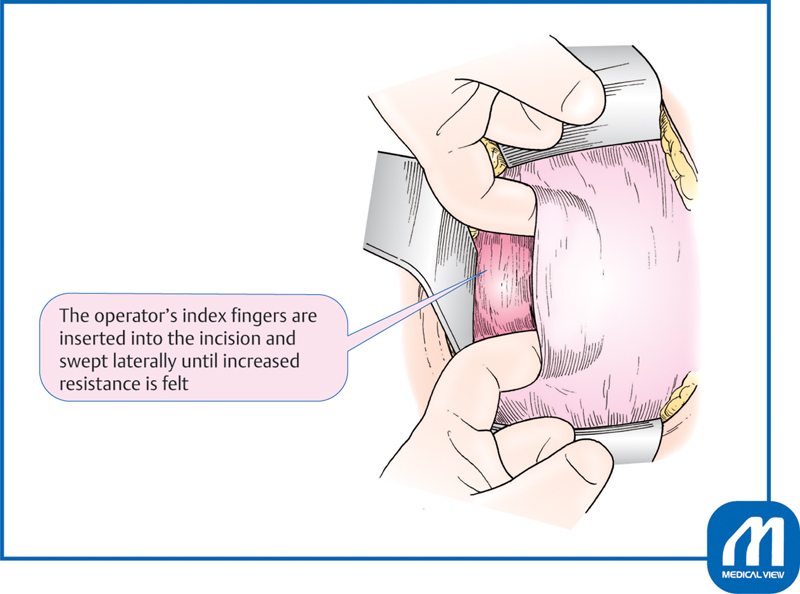
Lower uterine transverse incision and extension of the incision ③. (Reproduced with permission of Hiramatsu Y. Lower-segment transverse cesarean section. In: Hiramatsu Y, Konishi I, Sakuragi N, Takeda S, eds. Mastering the Essential Surgical Procedures OGS NOW, No.3. Cesarean Section (Japanese). Tokyo: Medical View; 2010:28–41. Copyright © Medical View).

Next, the operator's index fingers are inserted into the incision and swept laterally until increased resistance is felt by the connective tissue of the side wall, blood vessels, and other structures ( Fig. 4 ). The operative wound may be developed by pulling the tissue up and down, but it is better to open the tissue to the left and right to understand the amount of force being used.

**Fig. 5:**
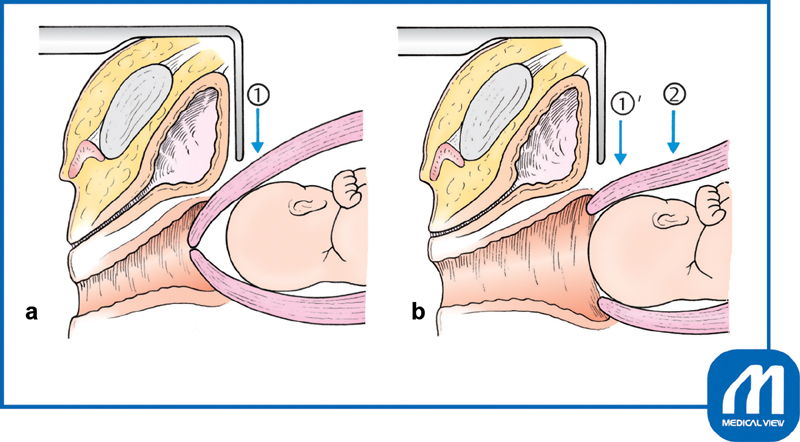
**(a)** Uterine cervix is closed. **(b)** Uterine cervix is dilated ≥5 cm. When the uterine ostium is closed, the site of incision is ①. When the uterine ostium opens, the cervix is extended and the correct incision site rises to the position ②. If ①′ is incised in the same way as when the uterine ostium is closed, it results in cutting the very lower part. (Reproduced with permission of Hiramatsu Y. Lower-segment transverse cesarean section. In: Hiramatsu Y, Konishi I, Sakuragi N, Takeda S, eds. Mastering the Essential Surgical Procedures OGS NOW, No.3. Cesarean Section (Japanese). Tokyo: Medical View; 2010:28–41. Copyright © Medical View).

### Delivery of the Fetus

Several methods are used for delivery, including manual delivery, the use of an obstetrical spatula, and the use of a soft vacuum cup. The author uses an obstetrical spatula. It is difficult to make extra tears, because the obstetrical spatula is thinner than the thickness of the hand, and use of a spatula is easier than the use of a vacuum cup.

When the head is pushed out, the face should be wiped downward and the amniotic fluid should be removed from the nasal cavity. When the shoulder in front is caught, delivery is made easy by lifting the uterine incision with a finger. When the fetal shoulders are pumped out, the finger should be placed on the axilla and the fetus should be pumped out diagonally and upward. Immediately after delivery, both edges of the incision may be grasped with the forceps. It is important to correctly hold the edges of the incision because strong bleeding often occurs from both edges of the wound. The fetal nasal cavity and mouth should be suctioned with a suction bulb to remove amniotic fluid and induce crying. The umbilical cord is cut, and the infant is given to the midwife.

**Fig. 6:**
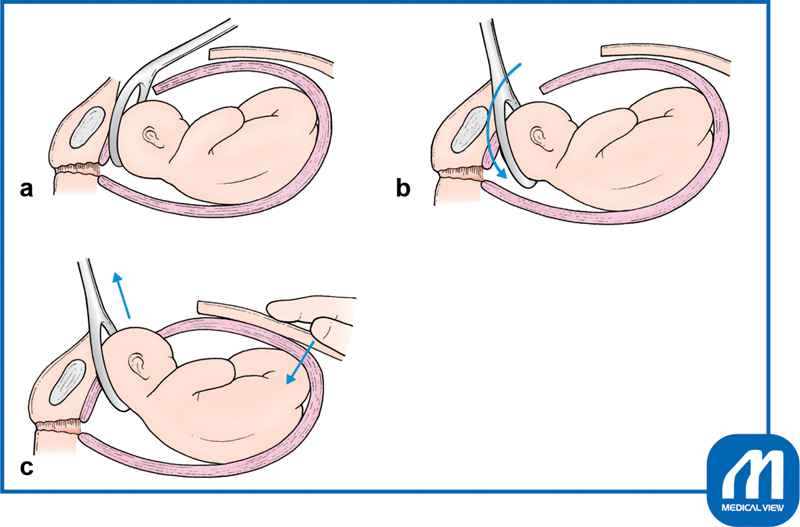
Delivery of the fetus: How to use obstetrical spatula. (Reproduced with permission of Hiramatsu Y. Lower-segment transverse cesarean section. In: Hiramatsu Y, Konishi I, Sakuragi N, Takeda S, eds. Mastering the Essential Surgical Procedures OGS NOW, No.3. Cesarean Section (Japanese). Tokyo: Medical View; 2010:28–41. Copyright © Medical View).

### Placental Delivery

At the same time as delivery, intravenous infusion of 5 units of oxytocin should start. While massaging the fundus of the uterus, the umbilical cord should be lightly pulled to easily deliver the placenta. The uterine cavity is manually wiped with gauze to remove any remaining placenta or membrane. This procedure is important because any remaining placental mass or fetal membrane may cause prolonged discharge of lochia, uterine atony, placental polyps, and other conditions.

When uterine contraction is poor, an additional 5 units of oxytocin should be injected into the myometrium.

### Closure of Uterine Incision 1 2 3 4 5

We use an absorbable polyglactin 910 suture (such as 0 Vicryl, CTB-1) to suture the uterine incision. This thread has a blunt needle tip, and there is no concern about tissue damage by accidental needle puncture. Additionally, the needle is large, making it suitable for suturing a uterine incisional wound. A two-layer suture may be used for the purpose of teaching surgical residents, but a one-layer suture may be used if there is no bleeding and the surface layers are fitted properly.

**Fig. 7:**
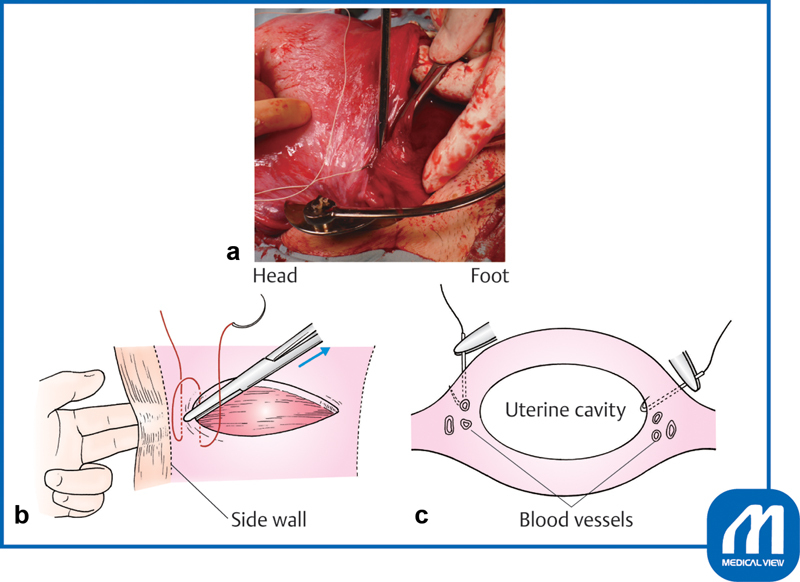
Closure of incision ①. **(a)** The wound edges are grasped with mucosal forceps or Pean forceps and pulled slightly inward, and a Z suture is placed to avoid slipping off the apex of the incision. **(b)** The surgeon should determine the margin of the uterine side wall. **(c)** The direction of the hand movement should not be straight down, but slightly inward at right angles to the myometrium to avoid vascular injury. (Reproduced with permission of Hiramatsu Y. Lower-segment transverse cesarean section. In: Hiramatsu Y, Konishi I, Sakuragi N, Takeda S, eds. Mastering the Essential Surgical Procedures OGS NOW, No.3. Cesarean Section (Japanese). Tokyo: Medical View; 2010:28–41. Copyright © Medical View).

Proper suturing of both edges of the incision is important. Because this region is particularly rich in blood vessels, it must be securely sutured. First, the wound edges are grasped with mucosal forceps or Pean forceps and pulled slightly inward, and a Z suture is placed to avoid slipping off the apex of the incision ( Fig. 7a,b ). This maneuver is performed because the blood vessel in the middle of the incision layer contracts and hides within the muscle layer; therefore, the surgeon must be sure to suture the blood vessel while pulling this part of the incision inward with forceps. If there is space for myometrial tissue outside the wound, the first suture should be made 5 mm outside the apex of the incision to avoid ( Fig. 7b ).

**Fig. 8:**
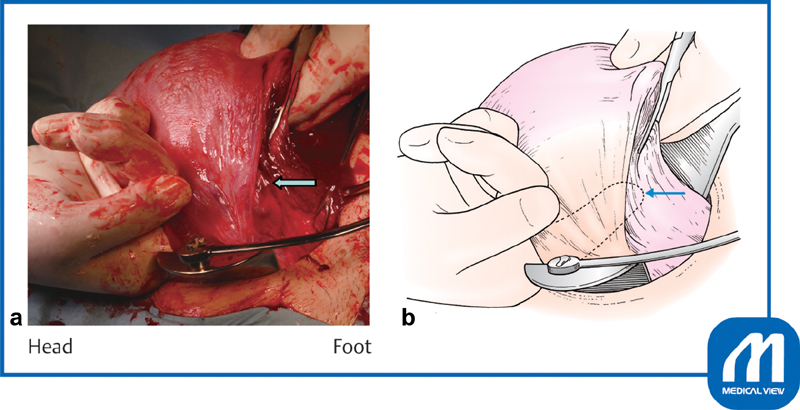
Closure of incision ②. The surgeon's finger should be placed under the broad membrane to determine the location of the uterine side wall. Arrow shows the location of the side wall margin. (Reproduced with permission of Hiramatsu Y. Lower-segment transverse cesarean section. In: Hiramatsu Y, Konishi I, Sakuragi N, Takeda S, eds. Mastering the Essential Surgical Procedures OGS NOW, No.3. Cesarean Section (Japanese). Tokyo: Medical View; 2010:28–41. Copyright © Medical View).

**Fig. 9:**
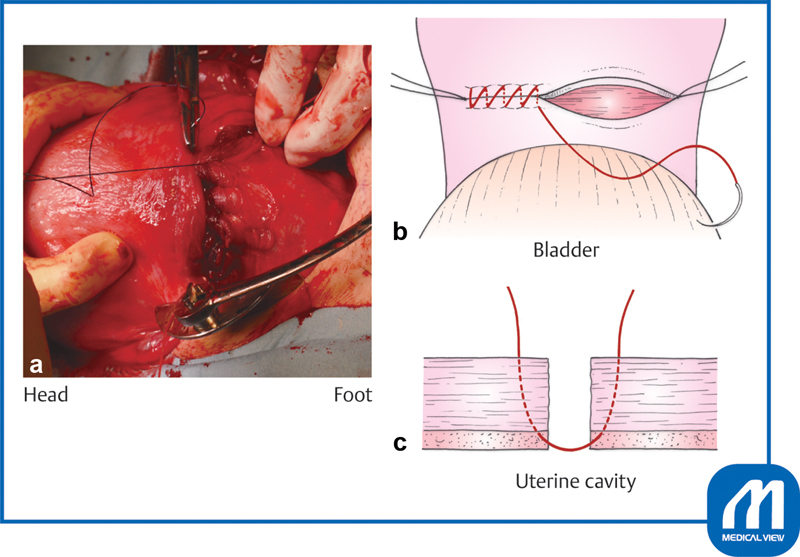
Closure of incision ③. The remaining portion of the incision is closed with running sutures. (Reproduced with permission of Hiramatsu Y. Lower-segment transverse cesarean section. In: Hiramatsu Y, Konishi I, Sakuragi N, Takeda S, eds. Mastering the Essential Surgical Procedures OGS NOW, No.3. Cesarean Section (Japanese). Tokyo: Medical View; 2010:28–41. Copyright © Medical View).

**Fig. 10:**
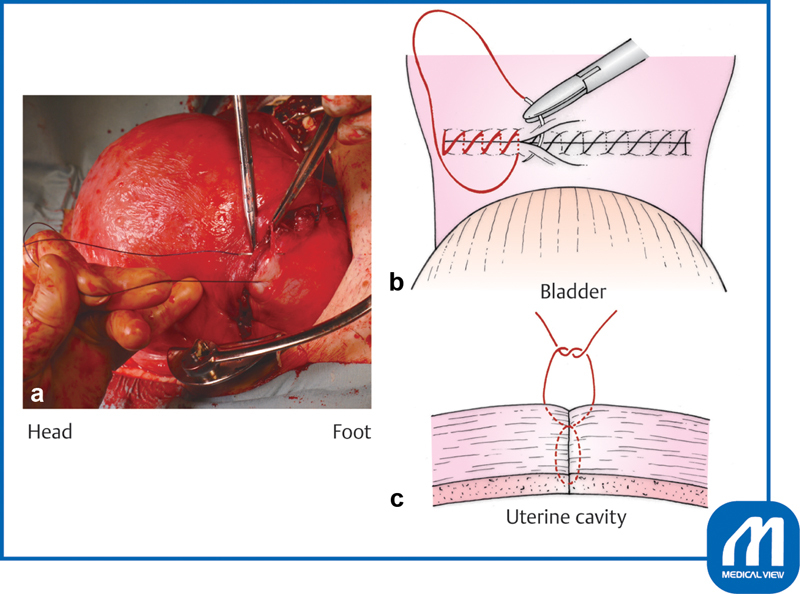
Closure of incision ④. The second suture should cover the first suture line.(Reproduced with permission of Hiramatsu Y. Lower-segment transverse cesarean section. In: Hiramatsu Y, Konishi I, Sakuragi N, Takeda S, eds. Mastering the Essential Surgical Procedures OGS NOW, No.3. Cesarean Section (Japanese). Tokyo: Medical View; 2010:28–41. Copyright © Medical View).

**Fig. 11:**
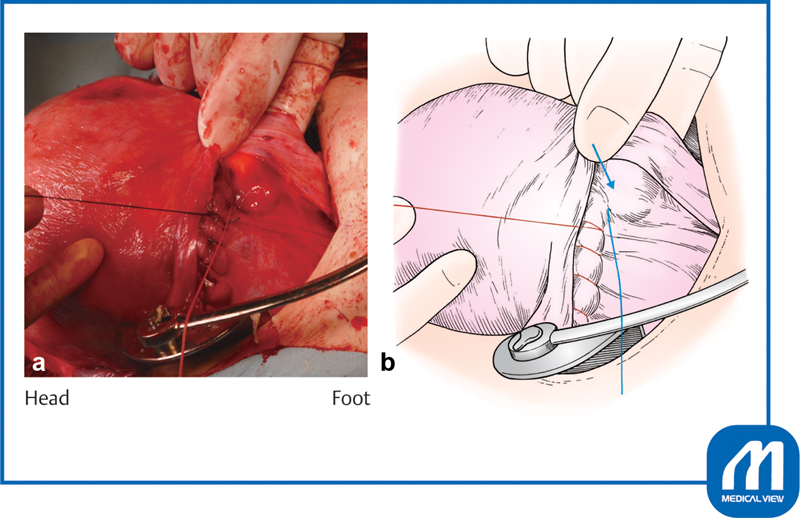
Closure of incision ⑤. It is also important to check the margin of the uterine side wall at the time of the second layer suture. (Reproduced with permission of Hiramatsu Y. Lower-segment transverse cesarean section. In: Hiramatsu Y, Konishi I, Sakuragi N, Takeda S, eds. Mastering the Essential Surgical Procedures OGS NOW, No.3. Cesarean Section (Japanese). Tokyo: Medical View; 2010:28–41. Copyright © Medical View).

If the cervical canal has not been dilated and dilation of the cervix is required, dilation of the cervical canal with Hegar dilators is performed at this time. The remaining portion of the incision is closed with running sutures ( Fig. 9 ). The second suture should cover the first suture line ( Fig. 10 ) after checking the margin of the side wall ( Fig. 11 ). If bleeding is present, the tissue should be clamped with a mosquito hemostatic forceps and ligated, or a Z suture should be added to stop the bleeding.

### Bladder Peritoneal Suture

Closure of the peritoneal bladder flap is not generally performed because there is no conclusive evidence that such closure has benefits. However, many surgeons use a synthetic absorbable antiadhesive film (Seprafilm, Interceed) to prevent adhesion. We usually suture the bladder peritoneum with 2–0 or 3–0 absorbable thread. We have not experienced problems at the time of repeated cesarean section when conventional bladder peritoneal sutures have been performed, and we have not used any antiadhesive film.

## Note

The myometrium may be sutured in one or two layers and in a interrupted or continuous pattern, and some reports have described the use of bladder peritoneal sutures. 6 7 In a survey of medical institutions in Japan, 8 bladder peritoneal sutures were placed to prevent adhesion in 69% of the institutions. With regard to bladder peritoneal sutures, a Cochrane review 9 showed that the operation time was shorter, percentage of patients with fever were lower, and hospitalization period was shorter in the nonsuture than suture group.

A systematic review and meta-analysis was to compare the effect of single- versus double-layer uterine closure on the risk of uterine scar defect. Women who received single-layer closure had a significantly thinner residual myometrium on ultrasound, however, no difference was found in the incidence of uterine dehiscence in a subsequent pregnancy. 10 11 There were no differences identified in risk of blood transfusion or other reported clinical outcomes. 11

### Irrigation of Abdominal Cavity and Closure of Abdominal Wall

The uterine cervix should be massaged from the front and back before closing the abdomen, and blood clots should be pressed into the vagina. The retractor is removed, the head of the operating table is raised, forceps are placed on the abdominal wall, and amniotic fluid and blood are aspirated and irrigated with warm saline. At this time, it is important to avoid strongly rubbing the surface of the uterus with gauze to prevent postoperative adhesion formation.

The surgeon should check whether the gauze counts match and then close the abdominal wall.

### Vaginal Disinfection and Abdominal Radiography

Finally, the vagina is cleaned to remove blood clots in the cervix and vagina. At this time, the assistant massages the uterine fundus to push out the blood clot in the uterine cavity. Finally, radiographs are taken to reconfirm that no gauze residue is present.

## Other Points to Note in Basic Cesarean Section 1 2 3 4 5

1. Precautionary points at repeated cesarean section
  As much information as possible about the previous delivery status and surgery method should be collected, even if the last delivery was performed at another hospital. Care should be taken during the peritoneal incision because adhesions may have occurred. Bladder dissection may be problematic if scarring is present from a previous cesarean section. Sharp dissection may be employed for this mobilization. Furthermore, at the time of uterine wall incision, the surgeon must exercise care to avoid damaging the fetus because the wall may become very thin. When the placenta is in the previous incisional wound, the frequency of adherent placenta is high; thus, very careful peeling and delivery are required.
  If the adhesions are extremely severe, it may not enter the abdominal cavity. In such cases, the bladder is lifted and the peritoneal and uterine walls are simultaneously incised. The position of the upper edge of the bladder must be confirmed by performing an ultrasound examination while the bladder is full.
2. Caution at breech presentation 4
  In the case of a frank breech presentation, the fetus is delivered in the same way as in a head presentation. In other words, a transverse incision is made and the hand or a cesarean spatula is used to deliver the fetal hip. Next, the fetus is held by fingers in the groin, and gentle figure-eight rotational traction is performed until the scapulas are clearly visible. The fetal arms and hands are delivered spontaneously during this maneuver in many cases. However, if an arm remains in the uterus, upward traction is placed upon the fetal feet and two fingers of the other hand are passed along the humerus until the elbow is reached; the two fingers are then swept down over the face. This maneuver is the same as the classic upper limb solution method during vaginal breech delivery. If the fetus is not large, the head may come out at the same time; however, if the head is difficult to pull out, the assistant can help by lifting the uterine incision at the site where the head is stuck. If difficulty is still encountered, the Veit–Smellie maneuver can be used. Calm and correct performance of the above operations is important during delivery.

In the case of complete/incomplete breech presentation or foot presentation, the ankle of one leg should be grasped and pulled from the incision, followed by the other leg. When both ankles have come out, they are gently pulled until the hips have been delivered. Delivery then proceeds in the same manner as in the frank breech presentation.

## Notes

Surgical Steps

1. Laparotomy (longitudinal incision/transverse incision)
  ↓
2. Bladder peritoneal incision and dissection of bladder
  ↓
3. Lower uterine transverse incision and extension of the incision
  ↓
4. Delivery of the fetus
  ↓
5. Placental delivery
  ↓
6. Closure of incision
  ↓
7. Bladder peritoneal suture (not required)
  ↓
8. Peritoneal lavage
  ↓
9. Closure of abdominal wall
  ↓
10. Vaginal disinfection

## Notes

Tips and Warnings 1 2 3 4 5

1. Caution in cases of premature rupture of membranes or oligohydramnios
  When premature rupture of membranes or oligohydramnios occurs, no amniotic fluid is present between the uterine wall and the fetus. This increases the possibility of damaging the fetus with a knife at the time of uterine wall incision.
2. Caution in cases of labor arrest or deep engagement of the fetal head
  When the cervix has opened 5 cm or more, especially when labor has stopped and the cervix is almost at full dilation, the fetal head is deeply engaged and the cervical muscle has become thin. Therefore, care is needed to avoid injuring the fetus at the time of transverse incision. In addition, attention must be paid to the uterine incision site. At this time, the site to be incised moves up considerably, resembling an incision near the upper body part ( Fig. 5 ). If the cervix has been extended, and if the incision is made downward as in the case of a closed cervical canal without labor pain, the incision will be performed at a very low site near the external cervical os.

## Notes

Tips and Warnings

1. Tips for delivery of fetal head using an obstetrical spatula 1 2 3 4 5
  With the operator standing on the left side of the patient, the operator's left hand is inserted into the uterine cavity while sliding along the surface of the fetal head. If the head is deeply engaged in the pelvis, the head should be moved upward near the incision wound and slightly bent toward the ceiling. The obstetrical spatula is then inserted between the operator's hand and the fetal head in the direction shown in Fig. 6a ; then slides along the rounded surface of the fetal head and is advanced to the position shown in Fig. 6b . Next, as shown in Fig. 6c , the assistant pushes the uterine fundus downward from the abdominal wall, and in coordination with this movement, lifts the obstetrical spatula obliquely upward and places the fetal head on the concave surface of the spatula. The fetus can be delivered smoothly when the surgeon operates the spatula with the sense of carrying out the fetal head on the obstetrical spatula. If the spatula is used as a lever, extra tears may occur around the incision and endometrium, and the fetal head may also be injured by the tip of the spatula.
2. Is the size of the abdominal incision appropriate?
  If the abdominal incision seems to be narrow at the time of delivery of the fetal head, delivery should be stopped and the abdominal wall should be lifted toward the ceiling with fingers, enlarging the abdominal incision to avoid damaging the fetus. If delivery is forced without this additional operation, the fetal head will be damaged.

## Notes

Tips and Warnings 1 2 3 4 5

1. The performance of cesarean section has increased, and the resultant increasing cases of cesarean scar defects, placenta previa, and adherent placenta have become problematic. To reduce these complications as much as possible, it is necessary to carefully suture the muscle layers and ensure correct alignment.
2. Care must be taken when there is not enough space outside the incision. The following points should be checked: (1) The surgeon's finger should be placed under the broad membrane to determine the location of the uterine side wall, and care must be taken to avoid placing a thread at a site without the myometrium ( Figs. 7a and 8 ). (2) The direction of the hand movement should not be straight down, but slightly inward at right angles to the myometrium to avoid vascular injury ( Fig. 7c ). If a suture is placed at a site without myometrium, the uterine vein, ureter, intestine, and other structures may be damaged. If the blood vessel at the wound edge slips out, bleeding will continue from this site after ligation, and if this site is to be sutured for hemostasis, blood vessel damage and hemorrhage often increase. Therefore, extreme care is needed to ensure that the suture and ligation at both edges are performed in a single operation.
